# Analysis of Pineapple Mealybug Wilt Associated Virus -1 and -2 for Potential RNA Silencing Suppressors and Pathogenicity Factors

**DOI:** 10.3390/v7030969

**Published:** 2015-03-05

**Authors:** Kishore K. Dey, Wayne B. Borth, Michael J. Melzer, Ming-Li Wang, John S. Hu

**Affiliations:** 1 Department of Plant and Environmental Protection Sciences, University of Hawaii, 3190 Maile Way, Honolulu, HI 96822, USA; E-Mails: kishore@hawaii.edu (K.K.D.); borth@hawaii.edu (W.B.B.); melzer@hawaii.edu (M.J.M.); 2 Hawaii Agricultural Research Center, Kunia, Honolulu, HI 96797, USA; E-Mail: mwang@harc-hspa.com

**Keywords:** PMWaVs, RNA silencing, suppressor, local RNA silencing suppressor, systemic RNA silencing suppressor, plant virus

## Abstract

Higher plants use RNA silencing to defend against viral infections. As a counter defense, plant viruses have evolved proteins that suppress RNA silencing. Mealybug wilt of pineapple (MWP), an important disease of pineapple, has been associated with at least three distinct viruses, *Pineapple mealybug wilt associated virus* -1, -2, and -3 (PMWaV-1, -2, and -3). Selected open reading frames (ORFs) of PMWaV-1 and PMWaV-2 were screened for their local and systemic suppressor activities in *Agrobacterium*-mediated transient assays using green fluorescent protein (GFP) in *Nicotiana benthamiana.* Results indicate that PMWaV-2 utilizes a multiple-component RNA silencing suppression mechanism. Two proteins, p20 and CP, target both local and systemic silencing in *N. benthamiana*, while the p22 and CPd proteins target only systemic silencing. In the related virus PMWaV-1, we found that only one of the encoded proteins, p61, had only systemic suppressor activity. Of all the proteins tested from both viruses, only the PMWaV-2 p20 protein suppressed local silencing induced by double-stranded RNA (dsRNA), but only when low levels of inducing dsRNA were used. None of the proteins analyzed could interfere with the short distance spread of silencing. We examined the mechanism of systemic suppression activity by investigating the effect of PMWaV-2-encoded p20 and CP proteins on secondary siRNAs. Our results suggest that the PMWaV-2 p20 and CP proteins block the systemic silencing signal by repressing production of secondary siRNAs. We also demonstrate that the PMWaV-2 p20 and p22 proteins enhanced the pathogenicity of *Potato virus X* in *N. benthamiana*.

## 1. Introduction

Pineapple mealybug wilt associated viruses (PMWaVs) belong to the genus *Ampelovirus* of the family *Closteroviridae* [1]. At least four different PMWaVs are associated with pineapple [2]. All the PMWaVs are transmitted semi-persistently by mealybugs in the genus *Dysmicoccus* [3,4]. Most of the viruses in the *Closteroviridae* were once thought to have similar replication and gene expression strategies based on work done with *Beet yellow virus* (BYV) [5], but recently it has been shown that *Grapevine leafroll-associated virus-*3 (GLRaV-3), the type member of the genus *Ampelovirus*, has unique gene expression strategies [6]. To better understand the complex etiology of mealybug wilt of pineapple (MWP), it is essential that the genomes of the PMWaVs be functionally characterized. 

In Hawaii, PMWaV-2 has an important role in the etiology of MWP, causing severe wilt symptoms and yield reductions in the presence of mealybug feeding, whereas infection by PMWaV-1 alone, with or without mealybug feeding, may cause yield reductions but does not produce wilt symptoms [4,7]. Phylogenetic analyses firmly embeds PMWaV-2 within the genus *Ampelovirus* where it is closely related to GLRaV-1 and GLRaV-3, and possesses the typical genome organization of other *Ampeloviruses* [8]. PMWaV-1 also has been assigned to this genus largely due to their common insect vectors and despite its considerably divergent genome organization [9]. The genetic differences between PMWaV-2 and PMWaV-1 or -3, which include the presence of several 3’-ORFs in PMWaV-2 that have no homology with other proteins and no known predicted functions, and the apparently different roles that PMWaV-1, 2- or -3 have in MWP symptom development, suggest the possibility that such ORFs at the 3’-terminus may encode RNA silencing suppressors. Moreover, several members of the family *Closteroviridae* have been shown to encode such suppressor proteins at their 3’-terminus of their genomes, which suggests that certain ORFs in the genomes of PMWaV-1 and PMWaV-2 might encode similar RNA silencing suppressors.

RNA silencing is a powerful defense mechanism that plants employ to combat virus infection [10]. To counteract this silencing, plant viruses have evolved to encode silencing suppressor proteins [11,12]. The molecular mechanisms by which these viral suppressor proteins interfere with plant RNA silencing are diverse. Some virus silencing suppressors thwart the host silencing machinery at the site of infection; this is referred to as suppression of local silencing [13]. Other suppressors allow viruses to spread to tissues far from the infection site; this process is known as suppression of systemic silencing [14]. The molecular mechanisms involved in these processes are distinct from one another. Some suppressor proteins, such as the P38 protein from carmoviruses, interact with Dicer-like proteins (DCL) of the RNaseIII family of enzymes, to prevent the cleavage of viral dsRNAs into 21 to 30 nt small RNAs (sRNA) [15,16]. Other suppressors, including p19 encoded by *Tomato bushy stunt virus* [17], HC-Pro from *Potato virus Y* (PVY) [18], and p21 from *Beet yellows virus* (BYV) [19] specifically bind to 21-nt sRNAs, but not to longer sRNAs [17,20,21,22,23]. Still other suppressors, such as the CP of *Turnip crinkle virus* (TCV), bind sRNAs of different sizes [23,24]. In the normal anti-viral response, these sRNAs are incorporated into an effector complex called the RNA Induced Silencing Complex (RISC) that then directs the viral RNAs to sequence-specific viral RNA degradation [11,25]. Plant tissues distant from the site of viral infection can also be primed to silence viral sequences by mobile silencing signals [26]. These signals move through the vasculature in a source to sink fashion and inhibit the spread of virus into recipient cells by sequence-dependent RNA silencing [27]. Plants also have an amplification mechanism that heightens the antiviral response by recruiting plant RNA dependent RNA polymerase (RDRPs) to produce secondary viral siRNAs from the source siRNAs, thereby targeting additional, contiguous viral RNA sequences for degradation [28,29,30]. This secondary amplification is believed to be fundamental to the systemic movement of the silencing signal [31]. There are diverse arrays of silencing suppressors that also target different host protein components. For example: the 2b protein of *Cucumber mosaic virus* (CMV) interacts with ARGONAUTE1 (AGO1), the core of the RISC [32]; the p38 protein of *Turnip crinkle virus* (TCV) targets DCL4 [33]; the *Cauliflower mosaic virus* (CaMV) p6 protein targets the DRB4 protein [34]; and the V2 suppressor of *Tomato yellow leaf curl virus* [35,36] targets the SGS3 protein, a cofactor of RDR6 that is involved in the secondary amplification of RNA silencing. 

In addition to the great diversity of their mechanisms of action [37,38,39,40], many viral proteins with suppressor activity have also been identified as pathogenicity or virulence factors in plants [37,41,42]. Such suppressors are responsible for symptom induction or may also enhance virus accumulation [43,44]. Examples of this type of suppressor include the CP, the CPm, and the p22 proteins of *Tomato chlorosis virus* (ToCV) [45], and the P1 protein of *Wheat streak mosaic* (WSMV) [46]. Many members of the family *Closteroviridae* encode multiple suppressors in their genomes, including *Citrus tristeza virus* (CTV) and ToCV [45,47]. The ORFs of these suppressors are all located at the 3’-ends of their virus genomes [5]. However, many members also possess a single suppressor protein such as p21 of *Beet yellows virus* (BYV) [19] and p19.7 suppressor of *Grapevine leafroll-associated virus*-3 (GLRaV-3) [48]. 

Here, we report that PMWaV-2 utilizes a multiple-component RNA silencing suppression mechanism. Two of these encoded proteins, p20 and CP, target both local and systemic silencing, whereas proteins p22 and CPd, target only systemic silencing. In the related virus PMWaV-1, we found no proteins that have local suppressor activities and only one encoded protein, p61, which has systemic suppressor activity. All of the identified suppressors of local silencing found in PMWaV-2 have relatively weak suppressor activities, but two of these identified suppressors, p22 and p20, were found to enhance the pathogenicity of *Potato virus X* (PVX) in an experimental host.

## 2. Materials and Methods

### 2.1. DNA Constructs

**Figure 1:**
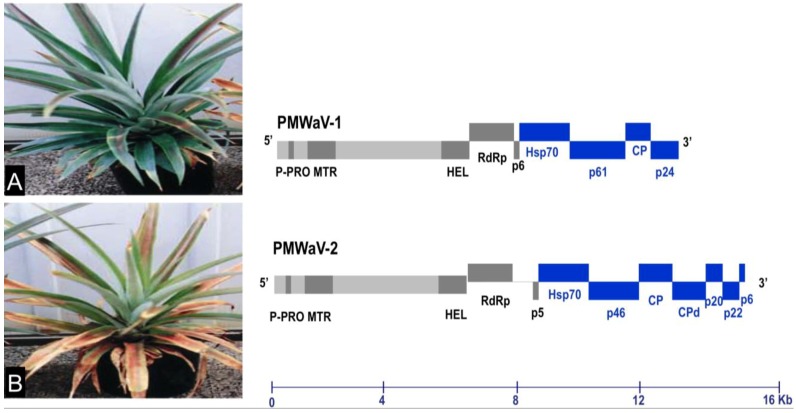
The genome organizations of PMWaV-1 and PMWaV-2. A symptomless pineapple plant infected by PMWaV-1 (**A**) and a symptomatic pineapple plant (**B**) infected by PMWaV-2 exhibiting typical wilting symptom are depicted alongside their genome organizations. Boxes represent open reading frames (ORFs), homologous genes, or domains are shown with the same pattern for both viruses. Blue boxes represent ORFs that were selected for screening of RNA silencing suppressor activity.

Positive-sense sGFP (pBIC/sGFP), dsGFP (inverted repeats; pBIC/dsGFP), and *Tomato bushy stunt virus* (TBSV) p19 (pBIC/p19) under the control of CaMV 35S promoter in binary plasmids [49], were gifts from Dr Kazuyuki Mise (Kyoto University, Kyoto, Japan). The sGFP gene in the constructs pBIC/sGFP and pBIC/dsGFP has 90% nucleotide sequence with the GFP gene (mGFP5) constitutively expressing in *N. benthamiana* line 16C. *Papaya ring spot virus* (PRSV) HCPro (pBIC/HCPro) was a gift from Yang Chen (University of Hawaii, Honolulu, HI, USA). To screen for potential local or systemic RNA silencing suppressors in PMWaV-1 and PMWaV-2, seven ORFs from the 3’-terminus of PMWaV-2 (Hsp70, p46, CP, Cpd, p20, p22, and p6) and four ORFs from the 3’-terminus of PMWaV-1 (Hsp70, p61, CP, and p24) (Figure 1) were individually amplified from total RNAs extracted from PMWaV-1 and -2 infected pineapple (*Ananus comosus*) plants by reverse transcription-PCR (RT-PCR) using specific primers that contained suitable restriction sites (Table 1), and cloned individually into the pGEM-T^®^-easy vector (Promega, Madison, WI, USA). All constructs were confirmed by sequencing (University of Hawaii Biotechnology-Molecular Biology Instrumentation Facility) and ligated into the binary vector pBIC between the 35S promoter and the nopaline synthase (NOS) terminator. Constructs were then electroporated into *A. tumefaciens* strain C58C1 with a Gene Pulser^®^ II system according to the manufacturer’s protocol (Bio-Rad, Hercules, CA, USA). To test for pathogenicity, selected ORFs from PMWaV-2 were inserted into the *Cla*I/*Sal*I site of the PVX vector pGR107 (Table 1), kindly provided by Dr. D.C. Baulcombe (University of Cambridge, Cambridge, UK), and were then individually introduced into *Agrobacterium* strain GV-3101 by electroporation as described previously. Transient assays for screening local suppressors were performed with 4-week old wild-type *N. benthamiana* plants. A total of 20 plants were agro-infiltrated with 4 plants in each pot used as a replication. Usually two well-expanded leaf from each plants were selected for agroinfiltration. The experiments were repeated twice.

**Table 1:** Primers used for PCR amplification of PMWaVs ORFs used to make expression constructs and to clone genes to produce Dig-labeled *in vitro* transcripts for Northern blot analyses. Random bases designated N (either A, T, G, or C) are included at the 5’-end of primers to increase the efficiency of restriction digestion.

Primers	Description or Sequence	Modifications
*Primers for constructing expression vectors*		
PMWaV-2 (HSP70) F	NNN*GGATCCA*TGGAAGTAGGATACGA	BamH1
PMWaV-2 (HSP70) F	NNN*GAATTC*CTATAGCCATCTCTTAC	EcoR-1
PMWaV-2 (P46) F	NNN*GGATCC*ATGCATCGCGAGTCCGC	BamH1
PMWaV-2 (P46) R	NNN*GAATTC*TTAAGTATTCGAACCAT	EcoR-1
PMWaV-2 (CP) F	NNN*GGATCCA*TGGCTCAGAATTACGTAGC	BamH1
PMWaV-2 (CP) R	NNN*GGTACC*CTACCCTGAAACAGCTC	Kpn-1
PMWaV-2 (Cpd) F	NNN*AGGCCT*ATGGAATTACAGCGGAT	Stu-1
PMWaV-2 (Cpd) R	NNN*GAATTC*CTAAACTCCATTATTTC	EcoR-1
PMWaV-2 (P20) F	NNN*GGATCC*ATGGAGTTTAGACCGAT	BamH1
PMWaV-2 (P20) R	NNN*GAATTC*TCATACTGGTATTTTGG	EcoR-1
PMWaV-2 (p22) F	NNNGGATCCATGAGTGAGGAGATCCTG	BamH1
PMWaV-2 (p22) R	NNNGGTACCTCATTTCTTACGACAGTTTCGG	Kpn-1
PMWaV-2 (P6) F	NNN*GGATCCA*TGAACACGAATGCTAA	BamH1
PMWaV-2 (P6) R	NNN*GAATTC*TTAATATTCATTTATAT	EcoR-1
PMWaV-1 (Hsp70) F	NNN*GGATCC*ATGGAGGTGGGTATTGATTT	BamH1
PMWaV-1 (Hsp70) R	NNN*GGTACCT*CACCTAACAATTTTGGAAT	Kpn-1
PMWaV-1 (p61) F	NNN*AGGCCTA*TGGCTTTGAGAGCAACTAG	Stu-1
PMWaV-1 (p61) F	NNN*GGTACC*TCACTGAGTGTGTTTTAATA	Kpn-1
PMWaV-1 (CP) F	NNN*AGGCCTA*TGGCTGATTCGAGCAAACA	Stu-1
PMWaV-1 (CP) R	NNN*GGTACCT*TAGCGTCCACCCATAA	Kpn-1
PMWaV-1 (P24) F	NNN*AGGCCT*ATGGAGAGGATTATATTGGT	Stu-1
PMWaV-1 (P24) R	NNN*GGTACCT*TAGATTTCAGATAGGATAC	Kpn-1
*Primers for constructing PVX vectors*		
PVX-PMWaV-2 (P20)F	NNN*ATCGAT*GGTAGCGACTCTGAGGTCTACAA	Cla-1
PVX-PMWaV-2 (P20)R	NNN*CCCGGG*CAGGATCTCCTCACTCATACTGGT	Xma-1
PVX-PMWaV-2 (P22)F	NNN*GTCGAC*CGCTATTAGACGCAACTATTCTGTTACC	Sal-1
PVX-PMWaV-2 (P22)R	NNN*CCCGGG*AGCATTCGTGTTCATTTCTTACGACA	Xma-1
PVX-PMWaV-2 (CP)F	NNN*ATCGAT*TCGTAGATTAAAGGCGATATGGCTC	Cla-1
PVX-PMWaV-2 (CP)R	NNN*GTCGAC*TTCTTCCTCCTACCCTGAAACAG	Sal-1
PVX PMWaV-2 (p20fs)F	GAGATCTCGACTGAAGTCGG	
PVX PMWaV-2 (p20fs)R	CCGACTTCAGTCGAGATCTC	
*Primers to clone genes for producing* in vitro *transcription templates*		
pTOPO (GFP)F	TTTCACTGGAGTTGTCCCAA	
pTOPO (GFP)R	GGCCATGGAACAGGTAGTTT	
PVX (CP)F	ATGTCAGCACCAGCTAGCACAACAC	
PVX (CP)R	TTATGGTGGTAGAGTGACAACAGCC	

### 2.2. RNA Isolation and Analysis

Total RNAs were isolated from *N. benthamiana* leaf tissues using TRIzol^®^ reagent as instructed (Invitrogen, Carlsbad, CA, USA). For Northern blot analysis of GFP and PVX mRNAs, 20 μg of total RNAs were separated on formaldehyde denaturing 1.25% agarose gels. The RNAs in the gel was transferred into Hybond^®^ N+ membranes (Roche, San Francisco, CA, USA) by overnight capillary transfer in 5× SSC/10 mM NaOH. For small RNA analysis, 20 μg of total RNAs were separated on 15% denaturing polyacrylamide gels containing 7 M urea in Tris-borate-EDTA (TBE) buffer. Following electrophoresis, gels were stained with ethidium bromide (0.5 μg/mL) to visualize RNAs. The RNAs were transferred to Hybond^®^ N+ membranes (Roche) by electroblotting in 0.5X TBE at 500 mA for 1 h. Membranes with transferred RNAs were UV-cross-linked at 1200 μJ in a UV Stratalinker^®^ 1800 (Stratagene, La Jolla, CA, USA). Membranes were stored at 4 °C until probed using digoxigenin-labeled RNA probes. These probes were prepared from *in vitro* transcription reactions with 1 μg cloned GFP fragment in pTOPO^®^ or PVX coat protein fragment in pGEM-T^®^-easy vector (Promega), and linearized using Maxiscript^®^ (Ambion, Grand Island, NY, USA) according to the manufacturer’s instructions. For GFP siRNA hybridizations, the digoxigenin-labeled *in vitro* transcription-derived RNAs were partially hydrolyzed in sodium carbonate buffer (120 mM Na_2_CO_3_; 80 mM NaHCO_3_) at 60 °C for 3 h and then neutralized with 3 M sodium acetate (pH 5.2) before use. The membranes were pre-hybridized in Ultrahybe-oligo^®^ (Ambion) for 1–2 h at 40 °C. All hybridizations were performed at 40 °C overnight. Post hybridization washes were as described previously [50].

## 3. Results

### 3.1. Screening the 3’-Proximal ORFs of PMWaV-1 and PMWaV-2 for Local RNA Silencing Suppressors

**Figure 2:**
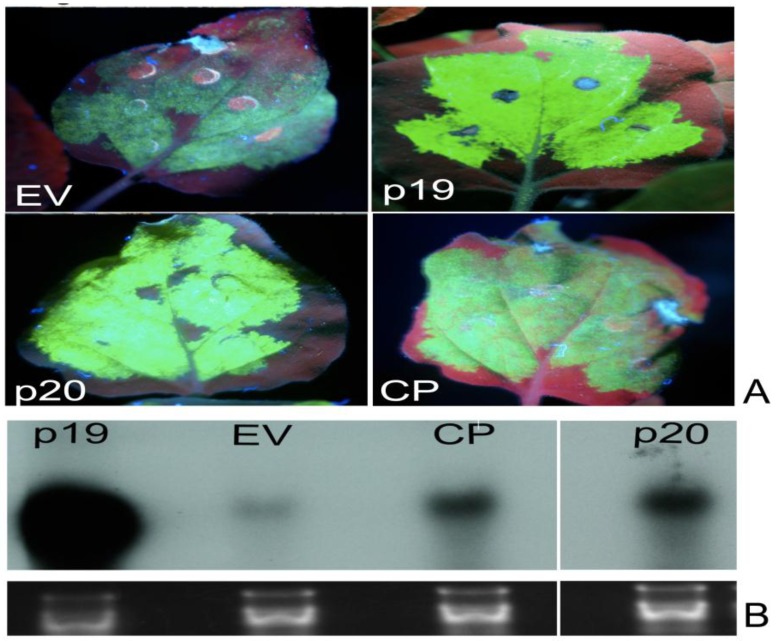

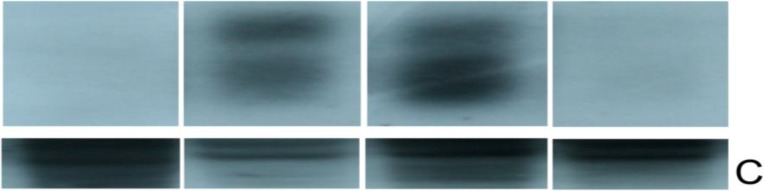
Identification of local suppressors in the genome of PMWaV-2. (**A**) WT. *N. benthamiana* plants were co-infiltrated with cultures of *Agrobacterium* carrying 35S-sGFP and *Agrobacterium* carrying individual constructs. Infiltrated leaves were examined under short-wavelength UV light and photographed with a Nikon 5000 digital camera at 5 days post-infiltration (dpi). Leaves co-infiltrated with 35S-GFP and pBIC-35S-empty vector (EV) or 35S-GFP with *Tomato bushy stunt virus* (TBSV)-35S p19 were used as negative or positive controls respectively. (**A**) shows fluorescence produced by the two identified local suppressors, p20 and CP. Northern blots of GFP mRNAs (**B**) and GFP siRNAs (**C**) from agroinfiltrated leaves at 5 dpi. Ethidium bromide staining of ribosomal RNA was used to confirm equal loading. Loading of RNAs for GFP siRNAs analysis was estimated by comparison to tRNAs on the same blot.

Seven ORFs from the 3’-terminus of PMWaV-2 (Hsp70, p46, CP, Cpd, p20, p22, and p6) and four ORFs from the 3’-terminus of PMWaV-1 (Hsp70, p61, CP, and p24) (Figure 1) were screened for potential local RNA silencing suppressors in agroinfiltrated *N. benthamiana* plants at 2, 3, 5, 7, 8, and 12 days post infiltration (dpi). As expected, two to three days following infiltration strong GFP fluorescence was observed in all leaves that had been co-infiltrated with *Agrobacterium* containing the 35S-empty vector and *Agrobacterium* containing 35S-sGFP (Figure S1) Similar results were observed in leaves co-infiltrated with *Agrobacterium* carrying 35S-sGFP and *Agrobacterium* carrying individual PMWaV-1 and PMWaV-2 ORFs (Figure S1). By 5 to 7 dpi GFP fluorescence declined significantly in those plants infiltrated with 35S-sGFP and 35S-empty vector, becoming almost undetectable by 7 dpi. As expected, co-infiltration with 35S-p19, the well-characterized RNA silencing suppressor of TBSV, elicited strong green fluorescence that persisted for several days. When leaves were infiltrated with a mixture of *Agrobacterium* suspensions containing both 35S-sGFP and constructs harboring various ORFs from PMWaV-1 and PMWaV-2, only ORFs p20 and CP from PMWaV-2 showed higher fluorescence in majority of the infiltrated leaves than did the empty vector controls at 5 dpi. None of the other ORFs from PMWaV-1 or -2 that were tested suppressed local GFP silencing (Figure 2). The p22 ORF of PMWaV-2, despite failing to suppress GFP local silencing, nonetheless caused the development of necrotic lesions in the infiltrated area of leaves, suggesting a potential role of p22 in pathogenicity that may be independent of silencing suppressor activity (Figure S2). The strong fluorescence observed when 35S-p19 was infiltrated persisted beyond 7 dpi and was maintained until 12 dpi. However when either the p20 or CP ORFs from PMWaV-2 were co-infiltrated with 35S-sGFP, there was a marked reduction of GFP fluorescence by 7 dpi that almost disappeared by 8 dpi (Figure S3). To confirm that the increased fluorescence of GFP was indeed due to the result of suppression of RNA silencing, the steady-state levels of GFP mRNAs were analyzed by Northern blots. The results obtained were consistent with the observations of GFP fluorescence (Figure 2); the GFP mRNA levels were significantly higher in leaves co-infiltrated with the 35S-sGFP and 35S-p20 or 35S-CP than in leaves co-infiltrated with the 35S-sGFP and 35S-empty vector, which showed significantly reduced GFP levels. This result indicates that p20 and CP of PMWaV-2 effectively delay RNA silencing in the transient-expression system. The effect of PMWaV-2 p20 and CP on local GFP silencing was considerably less than that of 35S-p19, as determined by the GFP mRNA levels at 5 dpi. Analysis of GFP siRNAs showed the accumulation of ~21 nt and ~24 nt siRNAs in leaves co-infiltrated with *Agrobacterium* carrying the 35S-sGFP and the 35S-empty vector, confirming that gene silencing was active in these plants [13,51]. In contrast, GFP-specific siRNAs were remarkably low in leaves co-infiltrated with *Agrobacterium* carrying PMWaV-2 p20 and were absent in leaves co-infiltrated with the 35S-p19 (positive control) (Figure 2). However, the siRNA levels produced by PMWaV-2 CP infiltrations did not show such a negative correlation; the levels of the GFP siRNAs were comparable to those produced by the negative control.

### 3.2. Effects of PMWaVs ORFs on the Short Distance Spread (10-15 cells) of the GFP Silencing Signal in N. Benthamiana 16C Plants

**Figure 3:**
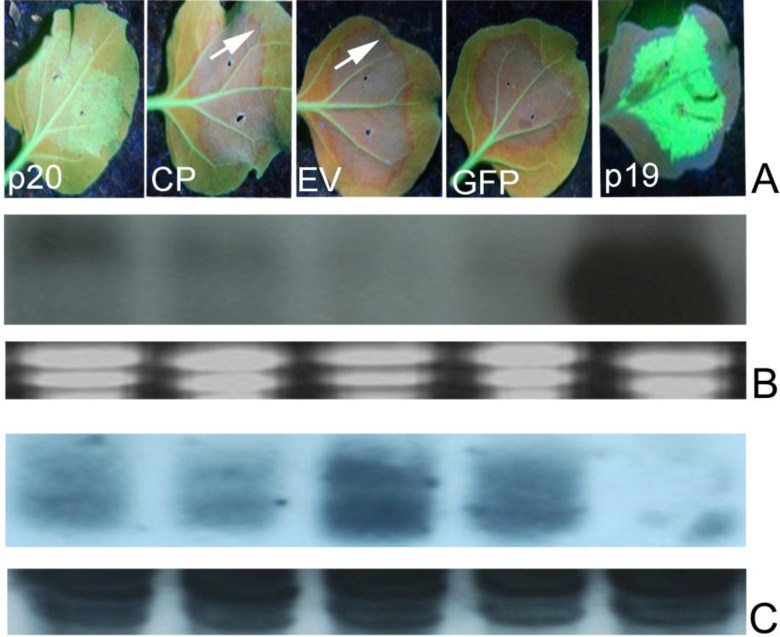
Effect of PMWaVs ORFs on the short distance spread (10–15 cells) of the GFP silencing signal in N. benthamiana 16C plants. (**A**) Leaves co-infiltrated with *A. tumefaciens* cultures harboring constructs pBI-35S-sGFP plus PMWaV-2 p20, CP, 35S-EV or 35S-p19. Photographs were taken at 7 dpi under short-wavelength UV light. White arrows indicate the red zone that indicates short-distance spread of the mobile RNA silencing signal at the edge of the infiltrated patch. Northern blot analysis of GFP mRNAs (**B**) and siRNAs (**C**) isolated from leaves infiltrated with the constructs as indicated. Loading of RNAs in GFP northerns were estimated by ethidium bromide staining of ribosomal RNA. Loading of RNAs in GFP siRNAs analysis was estimated by comparison to tRNAs on the same blot.

Following local silencing in the infiltrated leaves, the spread of the short-range silencing signal results in the loss of GFP transgene expression in a zone of 10–15 adjacent cells through the cell-to-cell movement of siRNAs [52,53]. This spread of silencing can be visualized under UV light in *N. benthamiana* 16C plants as a narrow red zone surrounding the infiltrated area, and reflects the silencing of the endogenous GFP transcripts constitutively expressed in *N. benthamiana* 16C plants. To investigate the effects of the ORFs of PMWaV-1 and -2 on this limited local spread, the formation of this red zone was monitored in *N. benthamiana* 16C plants after the constructs were co-infiltrated with 35S-sGFP (Figure 3). These red zones were observed in leaves co-infiltrated with 35S-sGFP plus 35S-empty vector by 7 dpi, whereas leaves co-infiltrated with 35S-sGFP plus 35S-p19 did not show any such red zone formation. P19 is known to block the short distance spread of the silencing signal [31]; we found that the red zone was not evident at 30 dpi during the 6 weeks observation period. Leaves infiltrated with 35S-sGFP and 35S-empty vector showed continuous expansion of the red zone during 7 to 12 dpi. The red zone was clearly visible by 7 dpi around the infiltrated area when 35S-sGFP plus PMWaV-2 CP was infiltrated, but when 35S-sGFP plus PMWaV-2 p20 was infiltrated there was a delay in the formation of the red zone (Figure 3) until 10 dpi that intensified by 12 dpi (Figure S4). All other PMWaV-2 ORFs that were tested developed this characteristic red zone by 7 dpi, similarly to the negative control (Figure S5). This result indicates that none of the PMWaV-1 or -2 proteins that were tested completely prevented the short-distance movement of silencing. PMWaV-p20 however, slightly delayed this short-distance spread of the GFP silencing signal. 

### 3.3. Effect of GFP dsRNA on PMWaV-2 p20 and CP Suppression of GFP Local Silencing

**Figure 4:**
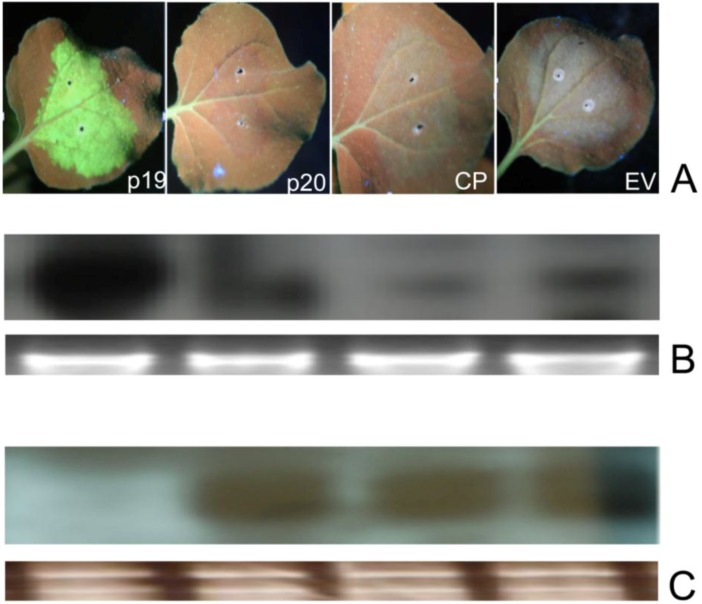
Effect of elevated levels of GFP dsRNAs on PMWaV ORFs on suppression of GFP local silencing. (**A**) Leaves co-infiltrated with A. tumefaciens cultures harboring constructs pBIC-35S-dsGF plus pBIC-35S-sGFP with PMWaV-2 p20, CP, 35S-EV, or 35S-p19 in WT *N. benthamiana*. 35S-p19 and 35S-EV are positive and negative controls respectively. Northern blot analysis of GFP mRNAs (**B**) and siRNAs (**C**) isolated from leaves infiltrated with the constructs depicted in the images. The images were photographed at 3 dpi. Loading controls for GFP mRNAs and siRNAs were estimated by ethidium bromide staining of ribosomal RNA.

RNAs that contain hairpin structures with extensive base paring have been found to be strong inducers of RNA silencing and are believed to be the signature molecules that a host plant recognizes to mount its antiviral RNA silencing effect against virus infection [54]. To determine if PMWaV-2 p20 and CP that were identified as local suppressors have the ability to interfere with GFP silencing triggered by a dsGFP inducer, *Agrobacterium* cultures containing 35S-sGFP and 35S-dsGFP together with either PMWaV-2 p20 or CP, or 35S-p19 and 35S-empty vector (OD^600^ = 0.5) used as positive and negative controls, were infiltrated into wild-type *N. benthamiana* leaves. At 3 dpi both PMWaV-2 p20 and CP constructs led to the complete disappearance of GFP fluorescence, while 35S-p19 showed retention of GFP fluorescence at high levels. Northern analysis of GFP mRNA levels correlated well with the expressed fluorescence levels (Figure 4). Further analysis of GFP siRNAs revealed the presence of characteristic ~21 and ~24 nt siRNAs when either PMWaV-2 p20 or CP were infiltrated, which was similar to that elicited by the 35S-empty vector control. No ~21–24 nt siRNAs were produced by infiltration with the 35S-p19 positive control (Figure 4). These results suggest that PMWaV-2 p20 and CP are not able to inhibit local silencing induced by a strong dsGFP trigger.

**Figure 5:**
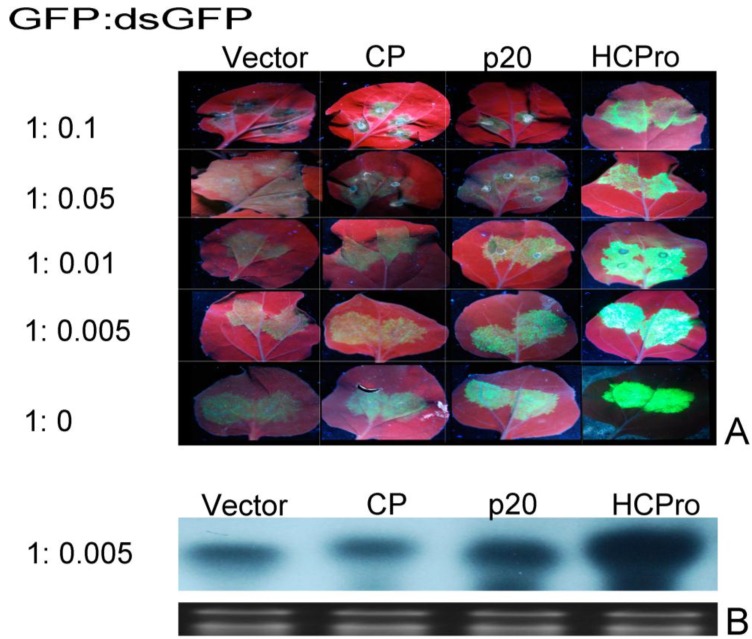
Effect of low levels of GFP dsRNA inducer on suppression of local GFP silencing by PMWaV-2 p20 and CP. (**A**) Silencing suppressor activity was assessed by agroinfiltration with mixed cultures of 35S-sGFP and 35S-PMWaV-2 p20 or 35S-PMWaV-2 CP together with decreasing concentrations of 35S-dsGFP in WT *N. benthamiana*. HCPro (PRSV) and pBIC-35S-EV were used as positive and negative controls respectively. Photographs were taken at 3 dpi under UV light. (**B**) Northern blot analysis for GFP mRNA levels in leaves agroinfiltrated with 35S-PMWaV-2 p20, 35S-PMWaV-2, 35S-sGFP, 35S-dsGFP at OD_600_ = 0.005. Loading controls for GFP northerns were estimated by ethidium bromide staining of ribosomal RNA.

To test whether PMWaV-2 p20 or CP have the ability to suppress silencing induced by low levels of dsRNA, dilutions of 35S-dsGFP inducer together with either PMWaV-2 p20 or CP, and 35S-sGFP were infiltrated into leaves of wild-type *N.benthamiana*. 35S-HCPro from PRSV was used as positive control. All *Agrobacterium* cultures except those containing 35S-dsGFP were adjusted to final OD^600^ = 1.0. Concentrations of 35S-dsGFP ranging from OD^60^ = 0.5 to 0.005 were prepared and used to co-infiltrate *N. benthamiana* according to the assay described by Martínez-Priego [55]. When 35S-dsGFP was used at OD^600^ = 0.1 or 0.05, neither PMWaV-2 p20 nor CP inhibited local silencing. However, when the 35S-dsGFP was diluted to OD^600^ = 0.01, PMWaV-2 p20 restored a low level of GFP fluorescence. The PMWaV-2 CP showed no such restoration of fluorescence, similar to that produced by infiltration with the empty vector. This effect suggests that PMWaV-2 p20 is not able to inhibit local silencing induced by high amounts of a strong dsGFP trigger but can suppress dsGFP triggered silencing at lower doses. When 35S-dsGFP was further diluted to OD^600^ = 0.005, an increase in GFP fluorescence in the presence of PMWaV-2 p20 was observed, demonstrating that PMWaV-2 p20 could effectively interfere with hairpin-induced silencing in the presence of low amounts of a strong inducer. To further confirm that the increased fluorescence of GFP seen at low 35S-dsGFP inducer levels was indeed due to the suppression of RNA silencing, the steady-state levels of GFP mRNAs were analyzed in Northern blots. The results were consistent with the observations of GFP fluorescence (Figure 5); the GFP mRNA levels were significantly higher in leaves co-infiltrated with 35S-sGFP, 35S-dsGFP, and 35S-p20 than in leaves co-infiltrated with the 35S-GFP, 35S-dsGFP, and 35S-CP or 35S-empty vector which showed reduced GFP levels. This confirms that 35S-p20 can inhibit the effects of low levels of hairpin inducer.

### 3.4. Screening for Systemic RNA Silencing Suppressors from the 3’-end Proximal ORFs of PMWaV-1 and PMWaV-2

Viral infection in plants can trigger transmission of RNA silencing over long-distances through the vasculature (systemic movement) [56,57]. To investigate whether any of the ORFs that were tested for local suppressor activity have the ability to inhibit systemic RNA silencing, GFP fluorescence was monitored in upper non-infiltrated leaves of *N. benthamiana* 16C plants infiltrated with *Agrobacterium* harboring either GFP alone (35S-sGFP) or with 35S-dsGFP in combination with individual ORFs from PMWaV-1 and PMWaV-2. The 35S-p19 silencing suppressor was used as positive control in these infiltration assays. Plants were visualized under UV light at 18 dpi, and the presence of systemic silencing, indicated by red fluorescence (due to chlorophyll) along the veins, was monitored (Table 2). It was found that plants infiltrated with PMWaV-2 CP plus 35S-sGFP had significantly higher numbers of plants that displayed GFP fluorescence (49 of 67) at 18 dpi (Pearson Chi-square *p* < 0.05). These plants maintained their GFP fluorescence, indicating suppression of silencing, for up to 30 dpi. When PMWaV-2 p20 was co-infiltrated with 35S-sGFP, 52% (33 of 63) of the plants displayed GFP fluorescence at 18 dpi (*p* < 0.05) that was maintained for up to 30 dpi (Table 2). In contrast, co-infiltration of 35S-sGFP plus the 35S-empty vector (negative control) resulted in only 33% (20 of 61) of the plants that had maintained GFP fluorescence. Chi-square analysis revealed that both PMWaV-2 p20 and CP had significantly higher suppression levels compared to the 35S-empty vector. All of the other ORFs of PMWaV-1 and PMWaV-2 displayed onset of systemic silencing at 18 dpi, which was not statistically different than produced by the empty vector. This silencing progressed over time and by 30 dpi was extensive (Table S1). The incomplete suppression observed in this study has also been reported for many viral systemic silencing suppressors that have been identified [58,59]. When the PMWaV-1 and PMWaV-2 ORFs were co-infiltrated with the hairpin-dsGFP construct, GFP fluorescence was lost in the majority of plants, which instead displayed only the red fluorescence due to chlorophyll in the upper leaves at 7 dpi. This was similar to the fluorescence produced by co-infiltration with the 35S-empty vector and 35S-dsGFP (Table S2) and was in contrast to the enhanced GFP fluorescence produced in plants infiltrated with 35S-p19. This result indicates that none of the PMWaV-1 or PMWaV-2 ORFs that were tested were able to suppress systemic silencing induced by 35S-dsGFP and also that only PMWaV-2 CP and p20 significantly inhibited systemic silencing induced by 35S-sGFP. 

To further test whether any of the PMWaV-1 or -2 ORFs could suppress systemic silencing by directly inhibiting the movement of silencing signals, we performed an assay as described by Guo and Ding [60], in which *Agrobacterium* containing constructs to be tested were infiltrated into transgenic *N. benthamina* 16C plants in upper (1) or lower (2) leaves of the same plant, or in proximal (3) or distal (4) zones of the same leaves as depicted in Table 3. 35S-p19 and 35S-empty vector were used as positive and negative controls respectively. Plants that were co-infiltrated with empty vector and 35S-sGFP, either in two different leaves of the same plant (treatments 1 or 2), or in the same leaf of a plant (treatments 3 or 4), exhibited systemic silencing after 14 dpi in most of the plants. In contrast, multiple ORFs from PMWaV-2 (CP, CPd, p22 and p20) and one ORF from PMWaV-1 (p61) showed suppression of systemic silencing, similar to 35S-p19, when either two different leaves or the same leaf (treatments 1 or 3) (Table 3) were infiltrated. However, when the same constructs were agroinfiltrated as in treatments 2 or 4, the majority of plants exhibited systemic silencing (Table 3). Infiltration in plants according to treatments 2 and 4 always resulted in systemic silencing and thus served as controls. Analyses by Chi-square was performed for all the ORFs in treatment 1 *versus* the 35% suppression efficiency of p20 from treatment 2; and all ORFs from treatment 3 *versus* the 42% suppression efficiency of p19 from treatment 4. The 35% and 42% suppression efficiencies were chosen as base values for this comparison because they were the treatments with the highest non-specific systemic silencing suppression percentage. Chi-square analysis using this approach showed a significant difference in suppression efficiency of several ORFs (*p* < 0.05). 

**Table 2:** Effect of PMWaVs ORFs on GFP-induced systemic silencing in transgenic N. benthamiana 16C plants. *Agrobacterium* carrying 35S-sGFP and individual PMWaV constructs were co-infiltrated with equal volumes of liquid bacterial cultures (OD^600^ = 1.0). 35S-sGFP and pBIC-35S-empty vector (EV) or TBSV-35S-p19 were used as negative or positive controls, respectively. The leaves were examined under short-wavelength UV light at 18 days post infiltration. Systemic silencing and suppression of systemic silencing is indicated in the photographs by red fluorescence developing as red trails and the lack of such red fluorescence in upper non-inoculated leaves respectively. Asterisks indicate significant differences in suppression efficiency between the individual constructs and the empty vector in Chi-square tests (*p* < 0.05).

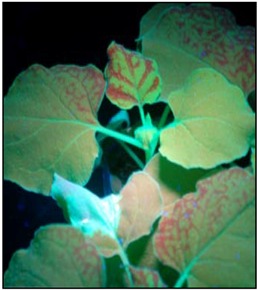		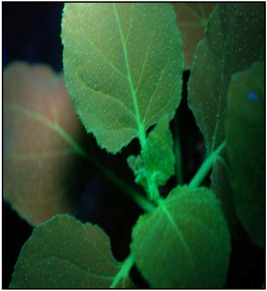	
Silenced		Not Silenced	
Virus	Gene/Construct	No. Plants Infiltrated	Suppression Efficiency (%)
	pBIC Vector	61	33
TBSV	P19	45	100*
PMWaV-2	Hsp70	40	22
PMWaV-2	P46	55	20
PMWaV-2	CP	69	74 *
PMWaV-2	CPd	50	30
PMWaV-2	P20	63	52 *
PMWaV-2	P22	64	36
PMWaV-2	P6	45	20
PMWaV-1	Hsp70	50	24
PMWaV-1	P61	60	30
PMWaV-1	CP	55	36
PMWaV-1	P24	45	18

**Figure 6:**
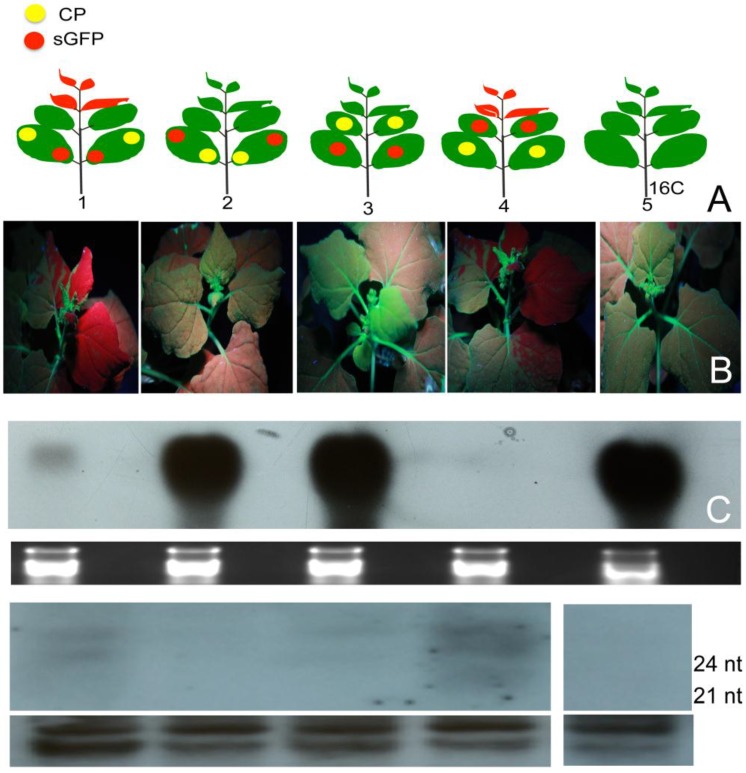
Molecular analysis of PMWaV-2 CP for its ability to interfere with the systemic RNA silencing signal. (**A**) Schematic showing simultaneous infiltration of 35S-sGFP (red circles) and PMWaV-2 CP constructs (yellow circles) in two different parts of the same leaf (proximal or distal; treatments 1 or 2) or into different leaves (lower leaf or upper leaf; treatments 3 or 4) of transgenic *N. benthamiana* 16C plants. Plant 5 is non-infiltrated plant. (**B**) Photographs of upper leaves showing the effect of the infiltrations described in panel (**A**). (**C**) Northern blot analysis of GFP mRNAs (upper) and siRNAs (lower) from non-infiltrated systemic leaves on infiltrated plants as depicted in panels (**A**) and (**B**). Loading of RNAs on GFP northerns was estimated by ethidium bromide staining of ribosomal RNA. Loading of RNAs for GFP siRNA analysis was estimated by comparison to tRNAs on the same blot.

PMWaV-2 CP completely suppressed systemic silencing (30 of 30 plants) and leaves maintained green fluorescence throughout the observation period. Northern blot analysis of GFP mRNA and siRNA levels in newly emerged leaves of plants infiltrated with only 35S-PMWaV-2 CP and 35S-GFP, either in the same leaf or in different leaves, and that also exhibited systemic suppression of silencing (plants 2 and 3; Figure 6) had increased GFP mRNA levels and decreased siRNA levels comparable to that of mock-infiltrated 16C plants (plant 5; Figure 6). In contrast, leaves exhibiting systemic silencing (plants 1 and 4; Figure 6) showed decreased GFP mRNA and increased siRNA levels.

**Table 3:** Interference of PMWaVs ORFs with the spread of systemic RNA silencing induced by GFP in transgenic *N. benthamiana* 16C plants. Schematic depicting simultaneous agro-coinfiltration of 35S-sGFP (red circles) and individual PMWaV ORFs (yellow circles) in separate leaves (lower or upper) of the same plant or in the same leaf (proximal or distal) of a single plant. Suppression of systemic silencing was indicated by the lack of red fluorescence in upper developing leaves as depicted in the figure. Results from the 35S-empty vector and 35S-p19 controls are sums from two experiments. All the remaining constructs were totals from single experiments. Asterisks indicate significant differences for treatments 1 and 3 when compared to the 35% suppression efficiency exhibited by p20 in treatment 2 and the 42% suppression efficiency exhibited by p19 in treatment 4 in Chi-square tests (*p* < 0.05). The 35% and 42% suppression efficiencies were chosen as base values for comparisons because they were the treatments with the highest percentage of non-specific systemic silencing suppression. 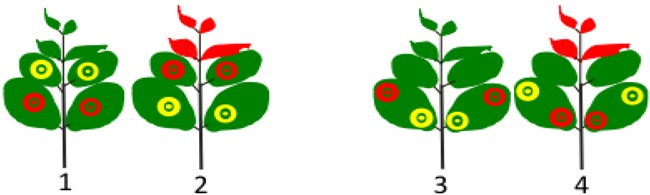

Virus	Gene		1	2		3	4
		No. Plants Infiltrated	Suppression Efficiency (%)	Suppression Efficiency (%)	No. Plants Infiltrated	Suppression Efficiency (%)	Suppression Efficiency (%)
	Vector	53	4	17	35	3	2
TBSV	p19	40	97 *	7	25	92 *	42
PMWaV-2	CP	28	100 *	7	28	100 *	42
PMWaV-2	p20	20	85 *	35	30	83 *	34
PMWaV-2	Hsp70	20	15	5	25	16	24
PMWaV-2	p22	24	83 *	29	24	79 *	25
PMWaV-2	p6	24	12	17	24	13	29
PMWaV-2	Cpd	20	60 *	6	25	84 *	28
PMWaV-2	p46	20	40	6	22	27	14
PMWaV-1	Hsp70	20	15	5	24	16	29
PMWaV-1	CP	16	37	19	16	38	31
PMWaV-1	p61	24	75 *	17	20	88 *	30
PMWaV-1	p24	24	41	25	22	18	32

### 3.5. PMWaV-2 p20 and CP have the Ability to Repress Accumulation of Secondary siRNAs

Systemic or long-distance movement of RNA silencing in plants depends upon the amplification of the siRNA silencing signal [56,57]. This amplification is carried out by host RNA-dependent RNA polymerase RDR6, which synthesizes new RNA molecules that extend beyond the initially targeted region in both the 3’- and 5’- direction. These newly-synthesized siRNAs are subsequently processed into secondary siRNAs, by DCL4 [61]. This phenomenon, known as transitivity [62], is responsible for the extensive movement of silencing [31].

**Figure 7:**
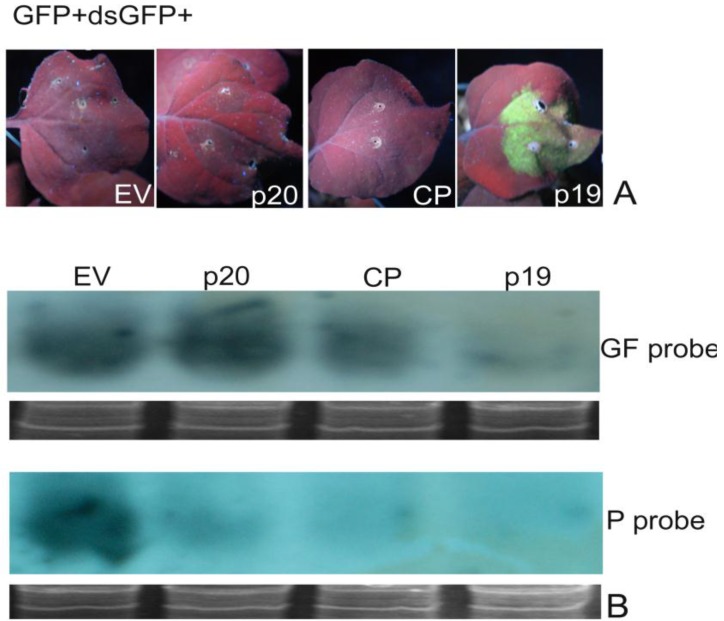
Effect of PMWaV-2 p20 and PMWaV-2 CP proteins on primary and secondary siRNA accumulation. (**A**) Leaves of WT *N. benthamiana* co-infiltrated with *A. tumefaciens* cultures harboring 35S-sGFP, 35S-dsGF, 35S-PMWaV-2 p20 or CP, 35S-EV, or 35S-p19. The 35S-p19 and 35S-empty vector (EV) are positive and negative controls, respectively. Photographs were taken at 6 dpi. (**B**) Northern blot analysis of GFP siRNAs from total RNA extracted from the infiltrated leaf at 6 dpi with either GF or P specific probes as indicated in the figure. Equal loading of mRNAs and siRNAs were estimated by ethidium bromide staining of ribosomal RNAs.

To determine if PMWaV-2 p20 and CP ORFs inhibit transitivity as described for other systems by Guo *et al.*, [63], leaves of wild-type *N. benthamiana* were agro-infiltrated with 35S-GFP and a 35S-dsGF construct comprising nucleotides 1-392 from the 5’-end of the GFP gene. Secondary siRNAs produced by transitivity arising beyond the dsGF portion were analyzed by Northern blots probed with the 3’-end of the GFP gene (“P” probe). TBSV-p19, which is known to inhibit transitivity, was agroinfiltrated with 35S-dsGF and 35S-sGFP as the positive control in all experiments. As expected, the leaf zones infiltrated with 35S-sGFP, 35S-dsGF, and 35S-p19 showed bright GFP fluorescence. However, leaves infiltrated with either PMWaV-2 p20 or CP together with 35S-sGFP and 35S-dsGFP displayed only the red fluorescence due to chlorophyll at 6 dpi, indicating that RNA silencing was occurring in these leaves (Figure 7). Northern blots probed with GF sequences from 35S-p20 or 35S-CP agroinfiltrated leaves showed accumulation of primary siRNAs similar to that of the negative control (empty vector). The 35S-p19, which is known to suppress both primary and secondary siRNAs, showed no such primary siRNAs accumulation. However, Northern blots of the same total RNAs from plants that had been agroinfiltrated with PMWaV-2 CP and p20 showed lower levels of secondary siRNAs when probed with the “P” sequence. These levels were similar to the levels shown with the positive control (35S-p19) indicating that transitivity was inhibited in these plants. Plants infiltrated with 35S-empty vector did not prevent the accumulation of these secondary siRNA.

### 3.6. Identification of Pathogenicity Factors by Expression of Selected PMWaV-2 ORFs from the Heterologous Potato Virus X Vector

To determine if any of the local suppressors identified from PMWaV-2 might also exhibit other effects, including increased virulence or alteration of symptom severity, PMWaV-2 p20, CP, and p22 were expressed in a heterologous system using a *Potato virus* *X* (PVX)-derived vector (pGR107). Although it failed to show local suppressor activity, PMWaV-2 p22 was included in this study because transient agroinfiltration with PMWaV-2 p22 consistently caused necrosis within the infiltrated zone of leaves (Figure S1). Furthermore, it has been reported that both p20 and p22 of PMWaV-2 showed homodimeric interaction to one another in yeast two-hybrid protein-protein interactions [64]. Such protein-protein interactions have been implicated in the regulation of viral pathogenesis in Closteroviruses [65]. 

**Figure 8:**
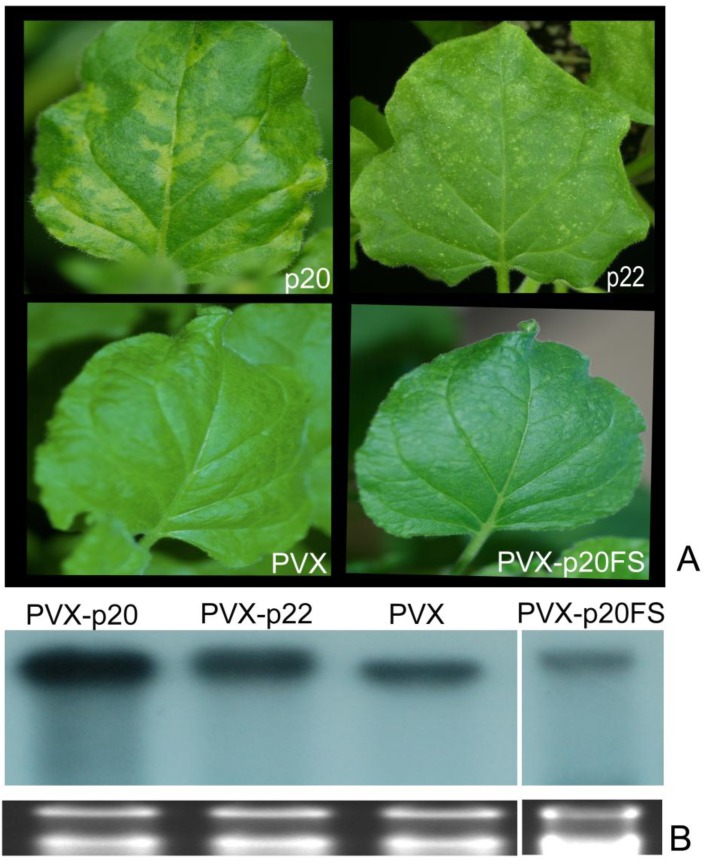
Effects of PMWaV-2 proteins on PVX pathogenicity. (**A**) Symptoms in systemic leaves of plants inoculated with PVX alone or with PVX containing PMWaV-2 ORFs p20, p22, or a frameshift mutant (FS) of PMWaV-2 p20. (**B**) Northern blot analysis with digoxigenin-labeled PVX coat protein RNAs of total RNAs isolated at 5 weeks post inoculation from upper uninoculated leaves of PVX-infected plants or plants infected with PVX containing PMWaV-2 p20, p22 ORFs, or a frame shift mutant (FS) of PMWaV-2 p20. Ethidium bromide staining of ribosomal RNA was used to confirm equal loading of RNAs.

Leaves of wild-type *N. benthamiana* (four-leaf stage) were infiltrated with PVX-p20, -CP, or -p22. PVX with no insert, and PVX-p20FS (frameshift mutant of p20) were used as controls. The plants were monitored from 12 dpi for the development of mosaic symptoms characteristic of PVX infection in non-inoculated systemic leaves. At 18 dpi, mild mosaic symptoms developed in many of the plants inoculated with PVX containing various PMWaV-2 ORFs (Table 4 and Figure 8). All plants that developed mosaic symptoms were tested with ELISA using antibodies against the CP of PVX (Agdia Inc., Elkhart, IN, USA) to confirm PVX infection. By 24 dpi, some of the PVX-PMWaV-2-p20 and -p22 inoculated plants, but not plants inoculated with PVX-PMWaV-2-CP, developed symptoms that were distinct from the typical mosaic symptoms induced by PVX (Table 4 and Figure 8). PVX-PMWaV-2-p20 induced mottling symptoms that were apparent after 24 dpi in all of the systemically infected leaves, whereas PVX-PMWaV-2-p22 induced chlorotic symptoms in a few of the systemically infected leaves after 24 dpi (Figure 8). The detection of PMWaV-2-p20 sequences by RT-PCR confirmed its presence in all of the systemically infected leaves of the PVX-PMWaV-2-p20 inoculated plants (data not shown). In contrast, PMWaV-2-p22 sequences could be detected only in those systemically infected leaves from PVX-PMWaV-2-p22 inoculated plants that displayed distinctive chlorotic spots, not in any of the other leaves that failed to develop these distinctive chlorotic spots but instead only showed the mild mosaic symptoms typical of PVX infection. Interestingly, the PVX construct could always be detected by RT-PCR in these leaves. This experiment was repeated three times with similar results. This suggests that the PMWaV-2-p22 gene had been eliminated from the PVX construct in those leaves of the inoculated plants that did not develop chlorotic spotting. 

**Table 4:** Plants displaying specific unique symptoms after infection with *PVX* containing selected PMWaV-2 ORFs. Results are means of three experiments (p20, p22, and CP), except for p20FS, which represents a single experiment. All plants that displayed PVX or gene-specific symptoms were also PCR positive for both the PVX and selected ORF sequences.

Description	p22	p20	CP	p20 FS	PVX
Number of plants with PVX symptoms/total number of plants	13/32	11/32	13/32	4/8	24/28
Number of plants with gene specific symptoms/total number of plants with PVX symptoms	3/13	7/11	0/13	0/4	-

Northern analysis of the RNA levels of PVX-PMWaV-2-p20 and PVX-PMWaV-2-p22 in symptomatic infected leaves was used to compare the RNA levels in leaves of PVX or PVX-PMWaV-2-p20 (FS) infected leaves. The incorporation of PMWaV-2-p20 and PMWaV-2-p22 into the PVX genome apparently enabled PVX to accumulate to higher levels in systemic leaves infected with the PVX-PMWaV-2-p20 or PVX-PMWaV-2-p22 constructs (Figure 8).

## 4. Discussion

Among the many plant diseases of economic importance, mealybug wilt of pineapple (MWP) is one of the most complex viral diseases for which the etiology is not well understood [2]. MWP is associated with a complex of plant viruses in the family *Closteroviridae*, and like other members of the *Closteroviridae*, the viruses associated with MWP have relatively large genomes. Plant viruses of the *Closteroviridae* have infection cycles that, from the time of initial genome expression to its replication, assembly, and cell-to-cell and systemic movement, are usually of relatively long duration [5]. Because of their relatively slow replication and infection cycles, it is thought that these large genomes must employ some multi-component and/or multi-level counter-defense mechanisms that protect them from attack by host RNA-silencing machinery [5]. One such strategy is the early synthesis of subgenomic RNAs that encode RNA-silencing suppressors to counteract host RNA silencing [5]. Other examples of mechanisms that protect the movement of virus particles throughout tissues of the host plant are the production of virus-encoded proteins, such as L-Pro and p20 of BYV, that act either as replication enhancers in the phloem, help to stabilize virions in the phloem, or guide virions through the plasmodesmata of neighboring cells [66,67].

Bioinformatic analysis of PMWaV-2 indicated that three ORFs (p46, p22, and p20) near the 3’-end of the genome have functions that are not known [9]. It is possible that, as in other *closteroviruses*, the products of these ORFs may be involved in suppression of RNA silencing. Using an *Agrobacterium* co-infiltration system in *Nicotiana benthamiana*, we have analyzed several 3’-ORFs of PMWaV-1 and -2 for their ability to suppress RNA silencing in this model host plant. Both the p20 and CP ORFs of PMWaV-2 were shown to have local and systemic suppression activity, while the p22 and CPd ORFs of PMWaV-2 were shown to suppress only systemic silencing. In contrast, none of the PMWaV-1 ORFs that were tested suppressed local silencing, but the p61 ORF was shown to have systemic suppressor function. We found that both the p20 and CP ORFs of PMWaV-2 were effective against (+) sense sGFP-induced silencing; their suppression effect diminished by 7 to 8 dpi, as evidenced by the disappearance of GFP fluorescence and the concurrent reduction of GFP RNA levels in infiltrated leaves. The PMWaV-2 p20 ORF effectively reduced the accumulation of GFP siRNAs until 5 dpi while the PMWaV-2 CP ORF did not cause such a reduction (Figure 2). Similar to the PMWaV-2 CP ORF, the p20 suppressor identified from *Citrus tristeza virus* (CTV) [47], p19.7 suppressor of *Grapevine leafroll-associated virus*-3 (GLRaV-3) [48] and the VP53, VP37, and the large capsid protein (LCP) suppressor identified from *Broad bean wilt virus 2* also do not prevent siRNA accumulation [68]. When the effects of PMWaV-2 p20 and CP were analyzed using a hairpin-GFP inducer, no suppression of GFP was found, as evident from the disappearance of GFP fluorescence, reduction of GFP mRNAs, and elevated levels of siRNA accumulation (Figure 4). The relatively weak suppressor activity that PMWaV-2 p20 displayed at low levels of hairpin-GFP inducer was completely nullified at higher hairpin-GFP inducer levels; this is similar to the suppression that is produced by the 16 K protein of *Tobacco rattle virus* (TRV) [55]. However, the p19.7 suppressor of GLRaV-3 is able to overcome strong silencing inducers such as dsRNA-GFP [48].

The local RNA silencing pathway may be targeted by PMWaV-2 p20 downstream of dsRNA conversion since the PMWaV-2 p20 ORF prevented siRNAs arising from sGFP but not dsGFP. PMWaV-2 p20 also inhibited silencing induced by low levels of hairpin-dsGFP, suggesting that this protein may also act at some point before the formation of dsRNAs. However, the complete mechanism remains to be elucidated. The silencing of (+) sense sGFP-RNA requires the conversion to dsRNA by the action of RDR6 [69,70,71], in concert with other co-factors, such as AGO1 [72],WEX [73], SDE3[31,74], and DCL4[52,75,76]. If PMWaV-2 p20 interacts with any of these cofactors at any step in the silencing pathway, suppression of hairpin-induced dsGFP silencing could occur. 

Suppressors such as p19 of TBSV and p21 of BYV protect target RNAs by reducing siRNA levels, thus preventing the formation of the 21–22nt RNAs that guide the endonucleolytic RNA-induced silencing complex (RISC) to degrade targeted RNAs [17]. The increased GFP expression levels that we observed following co-infiltration with the PMWaV-2 CP ORF, without the simultaneous reduction of siRNA accumulation, suggests that PMWaV-2 CP might exert its action downstream of siRNA production, possibly through destabilization of AGO1 [77,78]. Similar modes of action have been reported for the P0 protein of phloem-limited poleroviruses that target AGO proteins, [78] or the 2b proteins of cucumoviruses that have dual modes of silencing suppression, either inhibiting siRNA by sequestration [79] or preventing RISC assembly by interacting with AGO1 [80]. 

All of the tested ORFs caused the development of a distinctive red zone surrounding the area of infiltration that was evident at 8 dpi, suggesting the inability of all these proteins to prevent short distance spread of the silencing signal (Figure 3). The PMWaV-2 p20 ORF however, did not form this distinctive zone until 10 dpi. Similar observations have also been documented for the P1^RY^ and P1^CF^ proteins of *Cocksfoot mottle virus* [81], where it was suggested that weak local silencing suppressors are able to delay the movement of siRNA to neighboring cells [82]. However, in contrast such ring formation were also exhibited for strong suppressors, for example the V2 protein of *Tomato yellow leaf curl China virus* [59]. 

Short (21–22nt) and long (25nt) siRNAs are involved in the cell-to-cell and long distance movement of silencing signals, respectively [13,31]. We analyzed the 21–22 nt and 25 nt siRNAs produced by agro-infiltration of PMWaV-2 ORFs in infiltrated leaves of transgenic 16C *N. benthamiana* plants at 8 dpi. Both sizes of siRNA accumulated to approximately the same levels (Figure 3), suggesting that none of the proteins tested inhibited the production of short or long siRNAs preferentially. However, when these plants were observed for silencing in upper uninoculated systemic leaves, only the PMWaV-2 CP and p20 OFRs suppressed systemic silencing induced by sGFP, but not when induced by a hairpin-dsGFP construct. All of the other PMWaV ORFs that were tested for suppression of sGFP or dsGFP induced silencing showed silencing patterns similar to that produced by the empty vector in which the majority of plants were silenced. Similar results have also been observed for the Pns10 protein of *Rice dwarf phytoreovirus* that has the ability to suppress systemic silencing induced by sGFP but not systemic silencing induced by dsGFP [83].

Four of the seven ORFs from PMWaV-2 and one ORF from PMWaV-1 were shown to block or interfere with the movement of the systemic silencing signal in *N. benthamiana*. Although the precise nature of the systemic silencing signals has not been determined, it is known that the signal/signals move cell-to-cell through plasmodesmata and systemically through phloem [27,84], and, furthermore, that the RNA silencing signals are amplified in a process known as transitivity that involves host proteins [31]. The identification of several proteins in PMWaV-1 and PMVaV-2 that have systemic silencing suppressor activity led us to investigate whether two of these ORFs, PMWaV-2 CP or p20, could inhibit silencing at the level of transitivity. The results suggest that both CP and p20 do inhibit transitivity by restricting the amplification of the silencing signal. The p6 protein of *Rice yellow stunt rhabdovirus* (RYSV) also suppresses systemic silencing through interference with the secondary amplification of the silencing signal by interaction with RDR6 [63]. 

Many viral proteins that were originally identified as determinants of pathogenicity or virulence factors have been later identified as suppressors of RNA silencing [37]. The fact that PMWaV-2 is closely involved in the induction of MWP symptoms, and the identification in this study of two suppressors encoded by PMWaV-2, prompted us to investigate the possibility that these suppressors may also be pathogenicity factors in MWP. The development of chlorotic and mottling symptoms that were distinct from the symptoms produced by PVX infection alone or by PVX-P20 FS following infiltration with PVX-p20 and PVX-p22, suggests that these proteins may be associated with pathogenicity and/or virulence. Furthermore, Northern blot analyses of nucleic acids isolated from infiltrated leaves showed higher levels of PVX RNAs in plants inoculated with constructs containing PVX-PMWaV-2 p20 and p22. Interestingly, PMWaV-2 p22 did not show suppressor activity in agroinfiltration assays, but the areas in leaves infiltrated with this ORF became necrotic (Figure S1), suggesting that this protein might play some other role in the host-pathogen interaction. Moreover, miRNA pathways may also be involved in this response. Many well-characterized suppressors such as HCPro of PVY and p69 of *Turnip yellow mosaic virus* have been shown to interfere with miRNA-mediated silencing pathways resulting in developmental abnormalities manifested by visible symptoms [20,41].

The comparative and systemic analyses for suppressors of RNA silencing encoded in the genome of PMWaV-2 have led to the identification of two weak suppressors of local silencing, CP and p20, and four proteins CPd, CP, p22, and p20 that have suppressor of systemic silencing function. PMWaV-1 was found to encode only one protein, p61, with systemic silencing suppressor activity, and no proteins with local suppressor activity. The results presented in this study support our hypothesis that the involvement of PMWaV-2, but not PMWaV-1, in the etiology of MWP [4] might be due to the suppressors and pathogenicity factors identified in PMWaV-2 that are absent in PMWaV-1. The different silencing suppressors present in the genomes of PMWaV-2 and PMWaV-1 might explain how viruses of perennial crops, such as pineapple, cause persistent viral infections that are not lethal to their hosts, while many viruses that infect herbaceous hosts and encode strong suppressors are often lethal to the host. The absence of any local suppressor activity in PMWaV-1, which only encodes a single protein that only has systemic suppressor activity, further supports our hypothesis that viruses encoding only weak systemic silencing suppressors might be favored in persistent infections that do not lead to plant mortality. It will be also interesting to find out whether homologous suppressor protein for PMWaV-1 and -2 variants from different geographic locations might also show different levels of activity as noted for the p19.7 RNA silencing suppressor of GLRaV-3 [85]. In GLRaV-3 a single amino acid substitution is suspected for affecting the suppressor activity among phylogenetic groups [85]. The absence of strong co-relation of wilting symptoms with PMWaV-2 in Australia [2] might explain how subtle differences at the amino acid level might have drastic physiological effects manifested in symptom induction.

## Supplementary Files

Supplementary File 1
