# Supplementary material for: Analysis of Pineapple Mealybug Wilt Associated Virus -1 and -2 for Potential RNA Silencing Suppressors and Pathogenicity Factors
Source: Viruses. 2015 Mar 5;7(3):969–95. doi: 10.3390/v7030969 (PMC4379557; doi:10.3390/v7030969)
Supplement: Supplementary File 1 [file viruses-07-00969-s001.pdf]

Supplementary Materials

Analysis of Pineapple Mealybug Wilt Associated Virus -1 and -2 for Potential RNA Silencing Suppressors and Pathogenicity Factors

Kishore K. Dey, Wayne B. Borth, Michael J. Melzer, Ming-Li Wang and John S. Hu

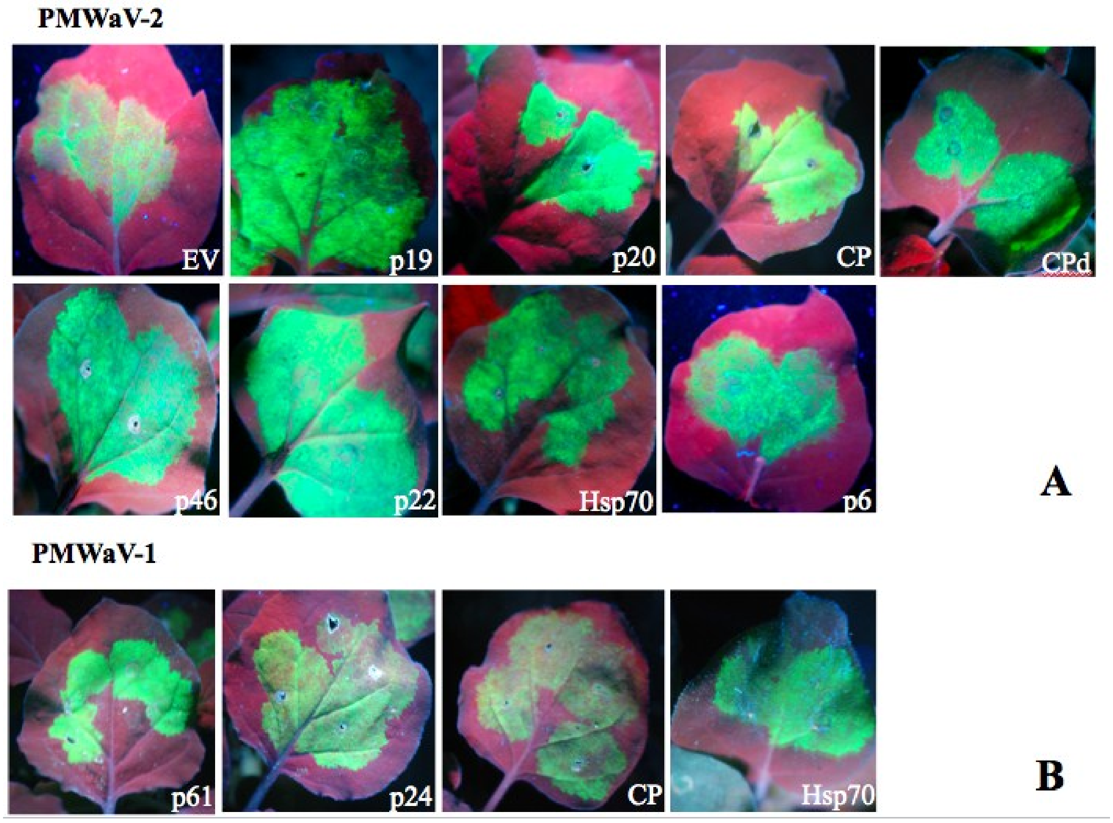

Figure S1. GFP fluorescence at 3 days post infiltration. WT. *N. benthamiana* plants were co-infiltrated with cultures of *Agrobacterium* carrying 35S-sGFP and *Agrobacterium* carrying individual PMWaV constructs. Infiltrated leaves were examined under short-wavelength UV light and photographed with a Nikon 5000 digital camera at 3 days post-infiltration (dpi). Leaves co-infiltrated with 35S-GFP and pBIC-35S-empty vector (EV) or 35S-GFP with *Tomato bushy stunt virus* (TBSV)-35S p19 were used as negative or positive controls respectively. (**A**) are PMWaV-2 constructs and (**B**) are PMWaV-1 constructs.

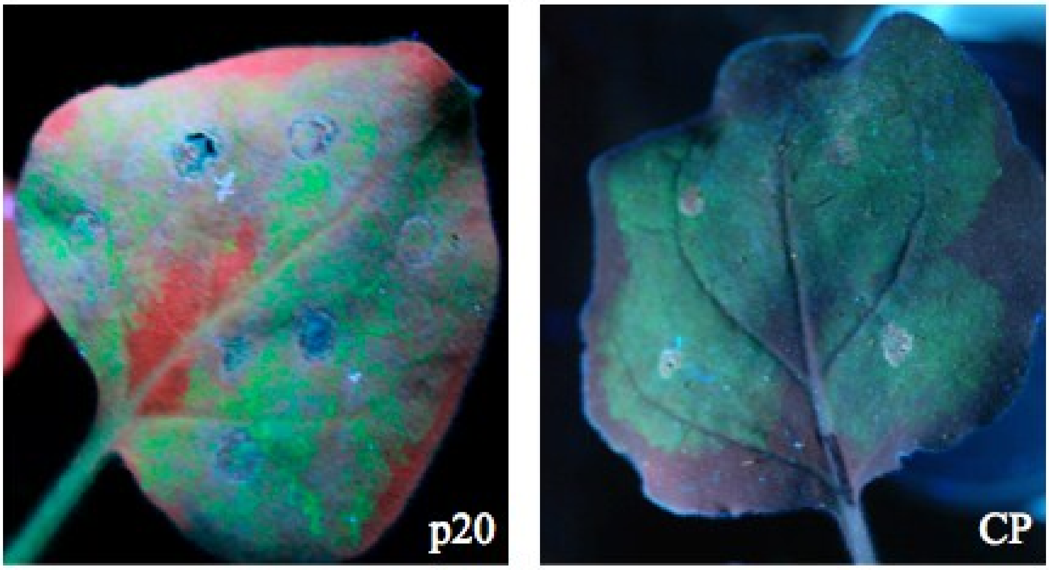

Figure S2. Decline of fluorescence by identified local suppressors. WT. *N. benthamiana* plants were co-infiltrated with cultures of *Agrobacterium* carrying 35S-sGFP and PMWaV-2 p20 and PMWaV-2 CP. Photograph shows decline of fluorescence produced by the two identified local suppressors, p20 and CP at 8 days post-infiltration (dpi).Infiltrated leaves were examined under short-wavelength UV light and photographed with a Nikon 5000 digital camera.

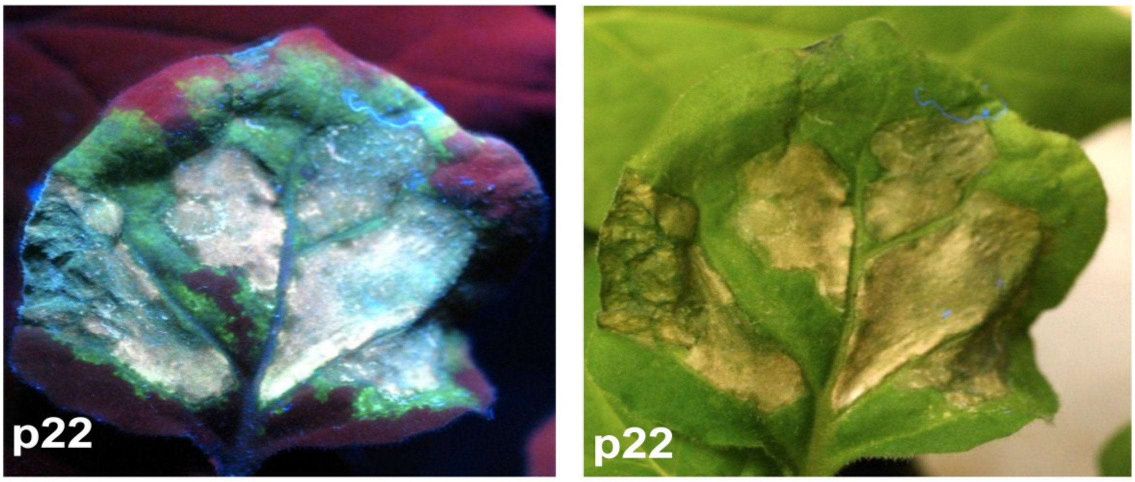

Figure S3. PMWaV-2-p22 showing necrosis. Leaves infiltrated with 35S-p22 and 35S-sGFP photographed under short-wavelength UV (Right panel) and under natural light (Left panel) show necrosis without local suppressor activity

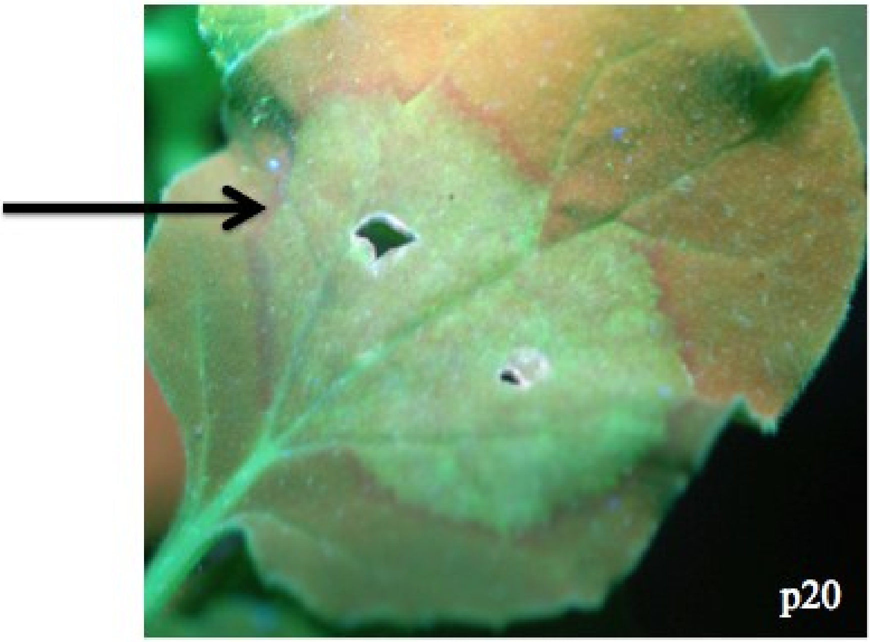

Figure S4. Formation of red zone due to short distance spread of GFP silencing by PMWaV-2 p20 at 12 days post infiltration.Leaves co-infiltrated with *A. tumefaciens* cultures harboring constructs pBI-35S-sGFP with PMWaV-2- p20. Photographs were taken at 12 dpi under short-wavelength UV light. Black arrow indicate the red zone that indicates short-distance spread of the mobile RNA silencing signal at the edge of the infiltrated patch.

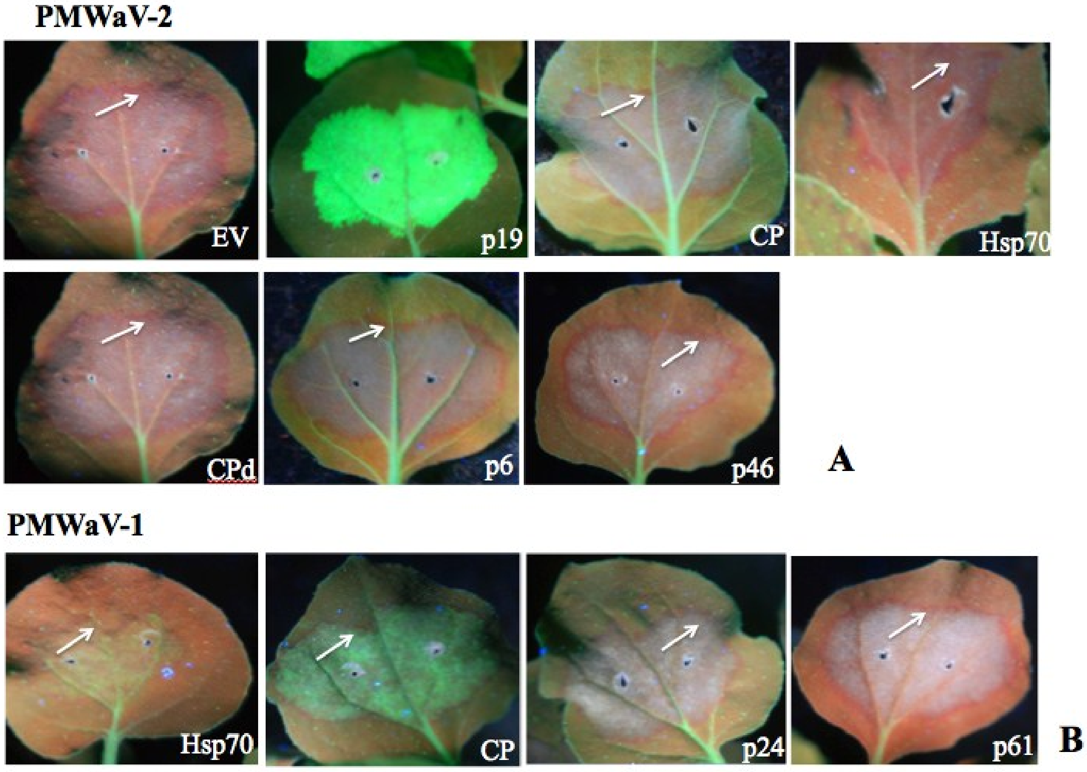

Figure S5. Effect of PMWaVs ORFs on the short distance spread (10–15 cells)of the GFP silencing signal in *N. benthamiana* 16C plants. (**A**) Leaves co-infiltrated with *A. tumefaciens* cultures harboring constructs pBI-35S-sGFP plus PMWaV-2 (CP, Hsp70, CPd, p6, p46), 35S-EV or 35S-p19 or (**B**) plus PMWaV-1 (Hsp70, CP, p24, p61),. Photographs were taken at 8 dpi under short-wavelength UV light. White arrows indicate the red zone that indicates short-distance spread of the mobile RNA silencing signal at the edge of the infiltrated patch.

Table S1. Effect of PMWaVs ORFs on GFP-induced systemic silencing in transgenic *N. benthamiana* 16C plants. *Agrobacterium* carrying 35S-sGFP and individual PMWaV constructs were co-infiltrated with equal volumes of liquid bacterial cultures ($OD^{600} = 1.0$). 35S-sGFP and pBIC-35S-empty vector (EV) or TBSV-35S-p19 were used as negative or positive controls, respectively. The leaves were examined under short-wavelength UV light at 4 weeks post infiltration. Suppression of systemic silencing was indicated by the lack of red fluorescence in upper non-inoculated leaves as shown in the figures in Table 2. Asterisks indicate significant differences in suppression efficiency between the individual constructs and the empty vector in Chi-square tests ($p < 0.05$).

**Virus**	**Gene/Construct**	**No. Plants Infiltrated**	**Suppression Efficiency (%)**
	pBIC Vector	61	13
TBSV	P19	45	100*
PMWaV-2	Hsp70	40	15
PMWaV-2	P46	55	12
PMWaV-2	CP	69	71 *
PMWaV-2	CPd	50	19
PMWaV-2	P20	63	50 *
PMWaV-2	P22	64	25
PMWaV-2	P6	45	14
PMWaV-1	Hsp70	50	15
PMWaV-1	P61	60	17
PMWaV-1	CP	55	14
PMWaV-1	P24	45	12

Table S2. Effect of PMWaVs ORFs on hairpin dsGFP-induced systemic silencing in transgenic *N. benthamiana* 16C plants. *Agrobacterium* carrying 35S-dsGFP and individual PMWaV constructs were co-infiltrated with equal volumes of liquid bacterial cultures (OD^{600} = 1.0). 35S-dsGFP and pBIC-35S-empty vector (EV) or TBSV-35S-p19 were used as negative or positive controls, respectively. The leaves were examined under short-wavelength UV light at 7 days post infiltration. Suppression of systemic silencing was indicated by the lack of red fluorescence in upper non-inoculated leaves as shown in the figures in Table 2. Asterisks indicate significant differences in suppression efficiency between the individual constructs and the empty vector in Chi-square tests ($p < 0.05$).

Virus	Gene/Construct	No. Plants Infiltrated	Suppression Efficiency (%)
	pBIC Vector	20	13
TBSV	P19	20	85*
PMWaV-2	Hsp70	18	15
PMWaV-2	P46	20	12
PMWaV-2	CP	20	10
PMWaV-2	CPd	20	19
PMWaV-2	P20	20	12
PMWaV-2	P22	18	11
PMWaV-2	P6	18	14
PMWaV-1	Hsp70	18	15
PMWaV-1	P61	16	17
PMWaV-1	CP	18	14
PMWaV-1	P24	20	12

© 2015 by the authors; licensee MDPI, Basel, Switzerland. This article is an open access article distributed under the terms and conditions of the Creative Commons Attribution license (http://creativecommons.org/licenses/by/4.0/).
